# *dSec16* Acting in Insulin-like Peptide Producing Cells Controls Energy Homeostasis in *Drosophila*

**DOI:** 10.3390/life13010081

**Published:** 2022-12-27

**Authors:** Ruo-Xin Zhang, Sha-Sha Li, An-Qi Li, Zhi-Ying Liu, G Gregory Neely, Qiao-Ping Wang

**Affiliations:** 1 Laboratory of Metabolism and Aging, School of Pharmaceutical Sciences (Shenzhen), Shenzhen Campus of Sun Yat-sen University, Shenzhen 518107, China; 2 The Dr. John and Anne Chong Laboratory for Functional Genomics, Charles Perkins Centre and School of Life & Environmental Sciences, The University of Sydney, Sydney, NSW 2006, Australia

**Keywords:** SEC16B, Sec16, *dSec16*, GWAS, obesity, food intake, triglyceride, starvation, insulin-like peptide producing cells

## Abstract

Many studies show that genetics play a major contribution to the onset of obesity. Human genome-wide association studies (GWASs) have identified hundreds of genes that are associated with obesity. However, the majority of them have not been functionally validated. *SEC16B* has been identified in multiple obesity GWASs but its physiological role in energy homeostasis remains unknown. Here, we use *Drosophila* to determine the physiological functions of *dSec16* in energy metabolism. Our results showed that global RNAi of *dSec16* increased food intake and triglyceride (TAG) levels. Furthermore, this TAG increase was observed in flies with a specific RNAi of *dSec16* in insulin-like peptide producing cells (IPCs) with an alteration of endocrine peptides. Together, our study demonstrates that *dSec16* acting in IPCs controls energy balance and advances the molecular understanding of obesity.

## 1. Introduction

Obesity, as a global health threat, increases the risk of diabetes, cardiovascular disease, hypertension and cancers [1]. The latest studies show that obesity leads to more severe symptoms in COVID-19 [2]. As of 2016, approximately 2 billion adults (39% of the world adult population) were overweight (body mass index (BMI) ≥ 25 kg m^−2^), and out of these, 671 million (12% of the world adult population) suffered from obesity (BMI ≥ 30 kg m^−2^) [3]. Considering the obesity pandemic and its threats to human health, there is an urgent need to reveal the pathogenic mechanism of obesity.

Obesity is caused by multiple factors from both environment and genetics. Functional loss of several key genes, including *Lep* [4], *LepR* [5] and *POMC* [6], causes severe obesity in humans. Studies of twins, family and adoption have demonstrated that obesity displayed a huge heritability between 40–70% [7]. Thus, the identification of genes underlying the development of obesity is fundamental to advancing our knowledge of this disease and to further helping us to formulate new effective strategies to curb obesity. Genome-wide association study (GWAS) is a powerful tool to uncover the associations between phenotypes and the corresponding genes. In past decades, a large number of GWASs have identified hundreds of genes that are associated with obesity, including FTO [8], MC4R [9] and *POMC* [10], and a few of them have been established as obesity genes before [3,11,12,13,14]. However, of the vast pool of obesity genes that GWASs have indicated, only a few have been functionally validated.

*Drosophila melanogaster* shares similarities with humans in the regulation of energy metabolism and possesses important organs and the endocrine system involved in energy metabolism. *Drosophila* has been shown to be a good model for studying obesity with conserved machinery of lipid metabolism and excessive fat accumulation as the main features in obese flies [15]. Moreover, it is conserved between *Drosophila* and humans for the majority of genes related to metabolic disease [16]. Additionally, *Drosophila* has numerous genetic tools, which are convenient to functionally validate obesity-associated genes proposed by GWAS studies [17]. In our previous studies, we have utilized *Drosophila* to functionally validate a group of genes revealed in different GWASs and have successfully identified novel regulators of energy homeostasis [18,19].

*SEC16B* has been indicated to be strongly associated with obesity in multiple studies with different populations [13,20,21,22]. Moreover, *SEC16B* is also associated with diabetes [23], metabolic syndrome [24] and bitter beverage preference [25] in GWAS studies. All suggest that SEC16B may play an important role in metabolism. So far, however, the physiological role of *SEC16B* in obesity or related metabolic diseases remains unknown.

SEC16B encodes a conserved peripheral membrane protein localized to endoplasmic reticulum (ER) exit sites (ERES), which is generally proposed to serve a mission in the biological process of protein secretion [26]. SEC16B participates in the COPII-coated vesicle coordinated anterograde transport of secretory elements exporting from the ER to Golgi, which is considered the first step of selective cargo export [27]. SEC16B acts as a scaffold in COPII formation, as well as a plays role as an element recruited to ERES by the COPII subunits [28]. On this basis, SEC16B is important for intracellular vesicle tracking. Evidence reveals that the central nervous system (CNS) senses and integrates central or peripherical signaling to regulate adipose stores, caloric intake and energy utilization [29]. In *Drosophila*, a lack of *dSec16* results in a blockage of protein transport from the ER, which possibly links to a deficiency of certain endocrine hormones [30]. It is speculated that SEC16B may be involved in the secretion of endocrine molecules, such as leptin [31], glucagon-like peptide-1 (GLP-1) [32], ghrelin [33], NPY and insulin [34], thus regulating energy homeostasis. Furthermore, *dSec16* is a *Drosophila* homolog of human SEC16B. Studies reported that *dSec16* was involved in nutritional stress responses [35,36]; however, little is known about *dSec16* playing a role in energy metabolism.

In this study, we functionally tested the functions of *dSec16* in the regulation of energy metabolism in *Drosophila*. We measured a few metabolic phenotypes in flies with RNAi of *dSec16* in whole-body or specific neurons. We found that *dSec16* functioning in insulin-like peptides producing cells (IPCs) has an impact on triglyceride (TAG) levels by modulating the endocrine peptides in *Drosophila*. Our results show that *dSec16* has a physiological role in the regulation of energy metabolism and, in particular, acts in IPCs.

## 2. Material and Methods

### 2.1. Fly Strains

Fly strains were maintained on a standard diet at 25 ℃, 12 light:12 dark cycles and 70% humidity. *Actin-GeneSwitch* (#9381), *Dilp2-Gal4* (#37516), *UAS-dSec16* RNAi (#53917), *Ok107-Gal4* (#854), *104y-Gal4* (#81014), *Dsk-Gal4* (#51981), *Dh44-Gal4* (#51987), *Lk-Gal4* (#51992), *OK371-Gal4* (#26160), *Chat-Gal4* (#6793), *55D01-Gal4* (#39110), *Ppk-Gal4* (#79278) and *Pdf-Gal4* (#6899) were purchased from the Bloomington Stock Center.

### 2.2. Diet Conditioning

The normal diet contains 1% agar, 2.45% yeast, 6.75% sugar, 6.25% corn flour, 1.2% *v/v* Nipigan (10% *w/v* in ethanol) and 0.6% *v/v* propionic acid. The high sugar diet contains 1% agar, 2.45% yeast, 20.25% sugar, 6.25% corn flour, 1.2% *v/v* Nipigan (10% *w/v* in ethanol) and 0.6% *v/v* propionic acid. For RU486 induction, male flies were collected the first day after hatch and transferred to normal food with 500 µM of RU486 (Sigma#M8046) for 6 days for induction. The 4–7-day adult male flies were used for experiments or transferred to HSD for further treatment.

### 2.3. Feeding Assay

Food intake was determined by a CAFE assay adapted from a previous study [37]. Five flies were housed in an empty vial with wet Kimwipes on the bottom. Liquid food was supplied to flies in 5 µL of capillaries through a homemade cap, which allows air exchange with the incubator. During the feeding assay, flies were fed a liquid food with 5% yeast extract (Merck #103753) and 10% sucrose. In all cases, food intake was measured over 24 h. Empty vials were used for evaporation controls. All food intake experiments were set up at zeitgeber time 6–8, and food intake was recorded exactly 24 h after the start of food loading. The length of food intake by flies was processed by Image J.

### 2.4. Triglyceride Assays and Body Weight Measure

Triglyceride assays were performed as in our previous study [38]. A total of 10 male flies were used for triglyceride assays and body weight. Body weight was assessed by analytical balance (Sartorius, with precision at 0.0001 g). For triglyceride assay, 10 male flies were homogenized in 200 μL dH_2_O on ice, then sonicated for 10 s using a probe sonicator on ice. After sonication, 800 μL ice-cold dH_2_O was added and mixed thoroughly. An amount of 50 μL of the mixture was used to determine the triglycerides, and 20 μL was used to determine the protein with the kits (Triglycerides kit, Nanjing Jiancheng; Modified Bradford reagent, BBI) under the manufacturer’s instructions. Triglycerides were normalized to protein.

### 2.5. Starvation Assay

Starvation survival was determined using the DAM (*Drosophila* activity monitor) (Trikinetics system), according to our previous study [39]. This system records a count when a fly crosses an infrared beam. Flies were loaded in DAM on 1% agar in 12-h light/dark cycles at 25 °C and monitored the activity every 60 min till the locomotor count reached 0. The survival numbers of flies at each time point were presented with a Kaplan–Meier curve drawn and compared by GraphPad Prism 9. Comparison between each genotype was processed by log-rank (Mantel–Cox) test.

### 2.6. Quantitative PCR

Total RNA was extracted by TRIzol, followed by extraction with chloroform and then precipitation with isopropanol. After washing with 75% RNAase-free ethanol, the quality of RNA is determined by NanoDrop^TM^ One. Reverse transcription was performed in an amount of 1 µg of mRNA using PrimeScript™ RT reagent Kit (TAKARA). RT-PCR was conducted with One-Step TB Green^®^ PrimeScript™ RT-PCR Kit (TAKARA), according to the manufacturer’s instructions. All primers used for qPCR are listed in Table S1. The reactions were run on Quantstudio 5 (Life technologies, Carlsbad, CA, USA).

### 2.7. Statistics

All statistical analyses were performed using GraphPad Prism 9. Pairwise comparisons were calculated by two-tailed unpaired *t*-tests. When evaluating the effect of genotype as a single independent variable among more than two genotypes, one-way ANOVA followed by Tukey’s post hoc test was used. Data are represented as mean ± SEM.

## 3. Results

### 3.1. Global Knockdown of dSec16 Increases Food Intake and TAG Levels

**Figure 1:**
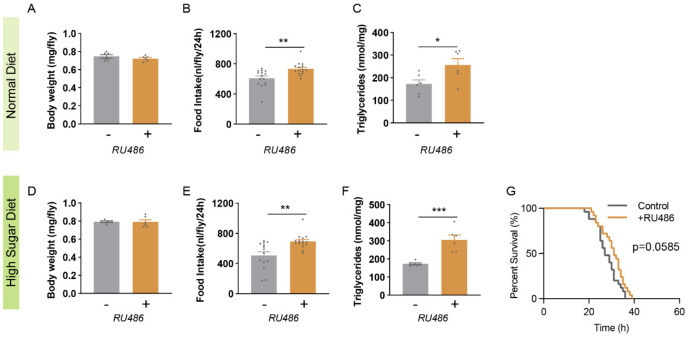
Globally inducible RNAi of *dSec16* increases food intake and triglyceride level in *Drosophila*. The globally inducible RNAi of *dSec16* was induced by RU486. (**A**) Body weight was not changed in *dSec16* RNAi flies (*Actin-GS-Gal4 >UAS-dSec16 RNAi*, *n* = 6). (**B**) Food intake (*n* = 15) and (**C**) TAG (*n* = 6) were significantly increased in *dSec16* RNAi flies. (**D**) Body weight (*n* = 6) was not influenced but (**E**) food intake (*n* = 15) and (**F**) TAG levels (*n* = 6) were increased in global *dSec16* RNAi flies under HSD. (**G**) Globally inducible *dSec16* RNAi flies were slightly resistant to starvation under HSD (*n* = 32 flies). *n*, indicates a biological replicate. Data are presented as mean ± SEM. Two-tailed unpaired *t*-test was used. The starvation assay was compared by log-rank (Mantel–Cox) test. * *p* < 0.05, ** *p* < 0.01, *** *p* < 0.001.

To ask whether *dSec16* plays a role in the regulation of energy metabolism in *Drosophila*, we used an inducible whole-body Gal4 driver (*Actin-GeneSwitch*, *Actin-GS*), specifically, RNAi of *dSec16* in adult flies *(Actin-GS-Gal4 > UAS-dSec16 RNAi)* by the induction by RU486. The mRNA expression of *dSec16* was reduced by 64% in the inducible RNAi flies (Figure S1). Firstly, we tested *dSec16* knockdown flies fed on a normal diet (ND). RU486 did not affect body weight (Figure S2A), food intake (Figure S2B) or triglyceride levels (Figure S2C). No difference was observed in body weight between the control group and the induction group (Figure 1A). However, food intake (Figure 1B) and TAG level (Figure 1C) were significantly increased in *dSec16* RNAi flies by RU486 when compared with the control flies without RU486 induction. Next, to mimic the high-fat diet in mouse studies, we subjected the inducible *dSec16* RNAi flies to HSD with RU486 induction for 14 days. Similarly, the *dSec16* knockdown flies did not show a change in body weight (Figure 1D) but did exhibit a significant increase in food intake (Figure 1E) and TAG levels (Figure 1F). Starvation survival is an important indicator of energy storage, and more fat means longer survival under starvation. As expected, *dSec16* knockdown flies survived longer than control flies under HSD (Figure 1G). These data indicate that *dSec16* knockdown causes food intake increase and fat accumulation in *Drosophila*.

### 3.2. Pan-Neuronal Knockdown of dSec16 Increases TAG Levels

**Figure 2:**
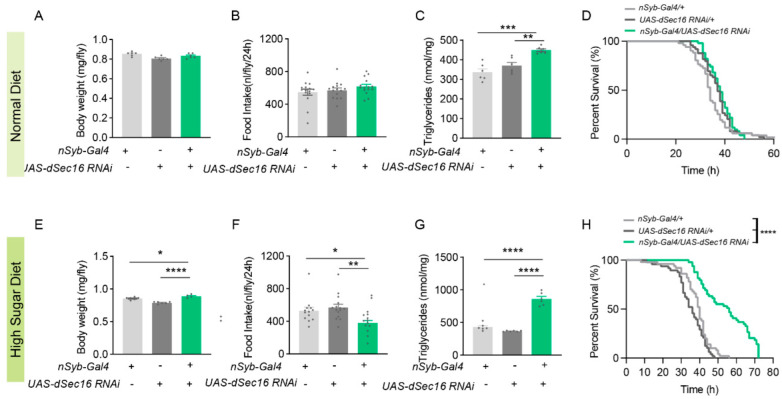
Pan-neuronal knockdown of *dSec16* increases TAG levels in *Drosophila*. (**A**,**B**) Body weight ((**A**), *n* = 6) and food intake ((**B**), *n* = 15) were not changed in pan-neuronal knockdown of *dSec16* (*nSyb-Gal4 > UAS-dSec16 RNAi*) flies when compared with control flies (*nSyb-Gal4/+* or *UAS- dSec16 RNAi/+*). (**C**) TAG was significantly elevated in *dSec16* RNAi flies (*n* = 6). (**D**) Starvation survival (*n* = 32 flies). (**E**–**G**). Body weight ((**E**), *n* = 6) and TAG ((**G**), *n* = 6) were increased but food intake was reduced ((**F**), *n* = 15) in pan-neuronal RNAi of *dSec16* flies (**H**) Pan-neuronal RNAi of *dSec16* flies showed more starvation resistance under HSD (*n* = 32 flies). *n*, indicates a biological replicate. Data are presented as mean ± SEM. One-way ANOVA with Tukey multiple corrections was used. The starvation assay was compared by log-rank (Mantel–Cox) test. * *p* < 0.05, ** *p* < 0.01, *** *p* < 0.001, **** *p* < 0.0001.

Since food intake is primarily controlled by the central nervous system, we reasoned that *dSec16* may function in the nervous system regulating metabolism. We then performed a pan-neuronal RNAi using *nSyb-Gal4*. Under ND, body weight (Figure 2A) and food intake (Figure 2B) were not significantly altered in pan-neuronal *dSec16* RNAi flies (*nSyb-Gal4 > UAS-dSec16 RNAi*) when compared with the parental controls (*nSyb-Gal4/+; UAS-dSec16 RNAi/+*). Consistent with whole-body knockdown, TAG levels were significantly increased in these pan-neuronal knockdown flies (Figure 2C); however, starvation survival of pan-neuronal knockdown flies was not significantly enhanced when compared with the parental control flies (Figure 2D). Next, we challenged these RNAi flies with HSD for 14 days. Surprisingly, HSD significantly decreased food intake (Figure 2E) but increased body weight (Figure 2F) and TAG levels (Figure 2G). As expected, these *dSec16* RNAi flies had longer starvation survival than the parental control flies (Figure 2H). Taken together, pan-neuronal knockdown of *dSec16* increases TAG levels under ND and HSD.

### 3.3. IPCs Specific-Knockdown of dSec16 Increases TAG Levels

**Figure 3:**
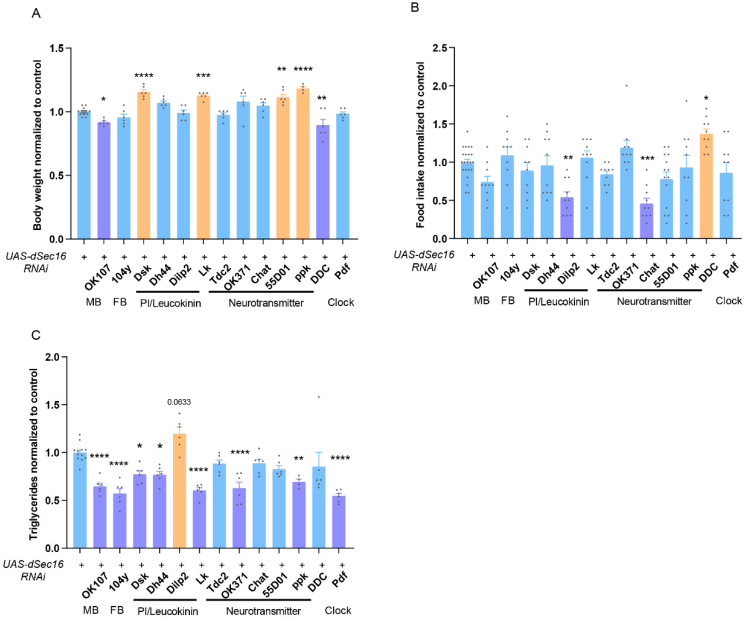
The RNAi of *dSec16* display differential effects on body weight, food intake, and TAG levels. (**A**–**C**) Body weight ((**A**), *n* = 6–12), food intake ((**B**), *n* = 10–20) and TAG ((**C**), *n* = 6–12) in flies with *dSec16* RNAi in various subsets of neurons belonging to mushroom body (MB), fan-shaped body (FB), pars intercerebralis (PI), leucokinin, neurotransmitter and clock. Results with significant decreases were presented in purple color but increases in orange color. *n*, indicates a biological replicate. Data are presented as mean ± SEM. One-way ANOVA with Tukey multiple corrections was used. * *p* < 0.05, ** *p* < 0.01, *** *p* < 0.001, **** *p* < 0.0001.

To identify the specific neurons in which *dSec16* may act, we have tested several subsets of neurons by crossing *UAS-dSec16 RNAi* flies with the corresponding Gal4 lines. When compared with the control *UAS-dSec16 RNAi*/+ flies, body weight was significantly decreased when *dSec16* was RNAi in fan-body neurons (*104y-Gal4*) and dopaminergic neurons (*DDC-Gal4*), but was significantly increased in drosulfakinin-producing neurons (*Dsk-Gal4*), leucokinergic neurons (*Lk-Gal4*), cholinergic neurons (*Chat-Gal4*) and sensory neurons (*Ppk-Gal4*) (Figure 3A). Interestingly, food intake was significantly decreased in flies with *dSec16* RNAi in IPCs (*Dilp2-Gal4*) and cholinergic neurons (*Chat-Gal4*) but was significantly increased in dopaminergic neurons (*DDC-Gal4*) when compared with the control *UAS-dSec16 RNAi*/+ flies (Figure 3B). Similarly, TAG levels were significantly reduced in flies with *dSec16* RNAi in mushroom-body neurons (*OK107-Gal4*), fan-body neurons (*104y-Gal4*), drosulfakinin-producing neurons (*Dsk-Gal4*), peptidergic neurons (*Dh44-Gal4*), leucokinergic neurons (*Lk-Gal4*), glutamatergic neurons (*OK371-Gal4*), sensory neurons (*Ppk-Gal4*) and clock neurons (*Pdf-Gal4*) (Figure 3C). Surprisingly, TAG levels were slightly increased in IPCs-specific *dSec16* RNAi flies. This is consistent with the results from pan-neuronal *dSec16* RNAi, suggesting that *dSec16* may act in IPCs to control fat accumulation.

**Figure 4:**
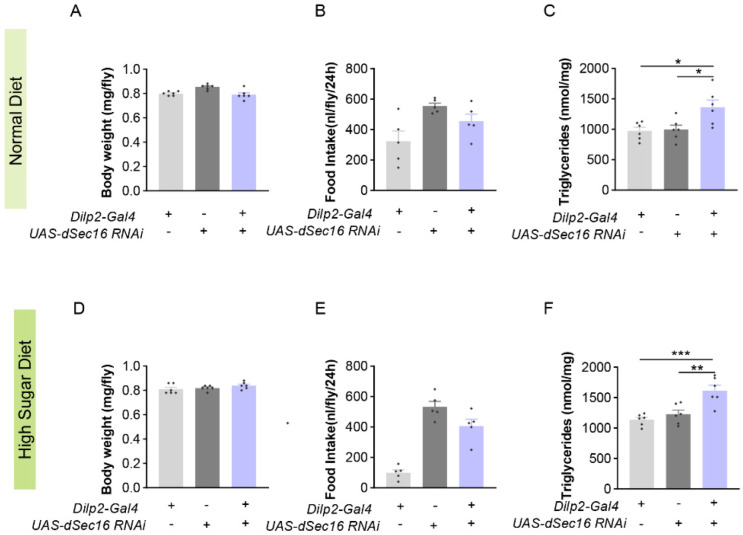
The RNAi of *dSec16* in IPCs increases TAG in *Drosophila*. (**A**–**C**) Body weight ((**A**), *n* = 6) and food intake ((**B**), *n* = 5) were not changed by RNAi of *dSec16* in IPCs (*Dilp2-Gal4 > UAS-dSec16* RNAi) but TAG ((**C**), *n* = 6) was significantly increased when compared with parental flies *(Dilp2-Gal4/+* or *UAS- dSec16 RNAi/+)* (*n* = 6). (**D**–**F**) Body weight ((**D**), *n* = 6) and food intake ((**E**), *n* = 5) were not changed but TAG was increased ((**F**), *n* = 6) in IPCs *dSec16* knockdown flies under HSD. *n*, indicates a biological replicate. Data are presented as mean ± SEM. One-way ANOVA with Tukey multiple corrections was used. The starvation assay was compared by log-rank (Mantel–Cox) test. * *p* < 0.05, ** *p* < 0.01, *** *p* < 0.001.

To further confirm the role of *dSec16* in IPCs, we generated flies with a specific RNAi of *dSec16* in IPCs. Body weight (Figure 4A) and food intake (Figure 4B) were not changed in IPCs-specific *dSec16* RNAi (*Dilp2-Gal4 > UAS-dSec16* RNAi) flies when compared with the parental controls (*Dilp2-Gal4/+; UAS-dSec16* RNAi/+). However, IPCs-specific *dSec16* RNAi significantly increased TAG levels (Figure 4C). Furthermore, consistent with the results from ND, similar results were observed for body weight (Figure 4D), food intake (Figure 4E) and TAG (Figure 4F) levels in IPCs-specific RNAi flies fed on HSD for 14 days. Both *dSec16* RNAi in the nervous system and in IPCs increase TAG, indicating that *dSec16* acting in IPCs controls fat accumulation.

### 3.4. dSec16 Influences Energy Homeostasis via Endocrine Peptides

**Figure 5:**
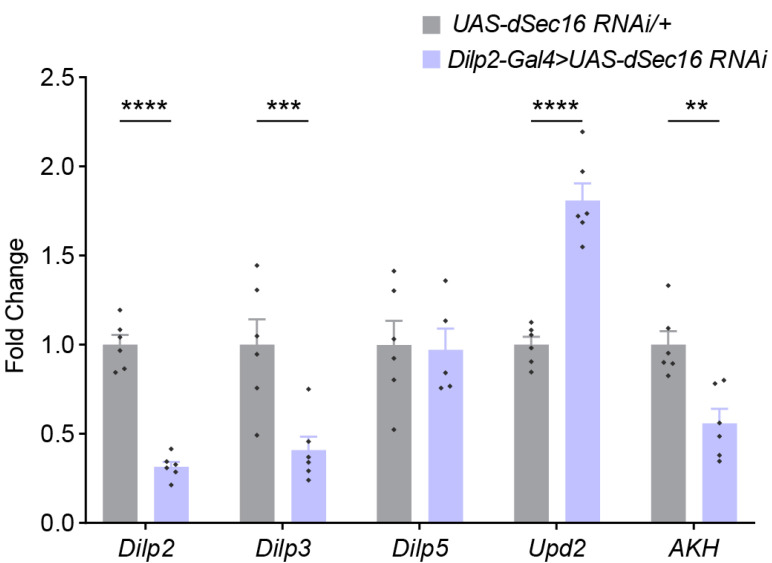
Inhibition of *dSec16* in IPCs impacts endocrine peptides. The mRNA levels for *dIlp2*, *dIlp3*, *dIlp5*, *Upd2*, *AKH* of flies with *dSec16* knockdown in IPCs (*Dilp2-Gal4 > UAS-dSec16* RNAi) with *UAS -dSec16 RNAi/+* line as control. *n* indicates a biological replication of 15 flies. Data are presented as mean ± SEM. Two-way ANOVA with Tukey multiple corrections was used. ** *p* < 0.01, *** *p* < 0.001, **** *p* < 0.0001.

IPCs regulate energy metabolism mainly through secretory molecules dIlps. IPCs can produce dIlp2, dIlp3 and dIlp5 [40]. To investigate if *dSec16* impacts fat accumulation by dIlps, we performed RT-PCR to determine the mRNA levels of these peptides. Of interest, the expressions of *dIlp2* and *dIlp3* was significantly less when *dSec16* was reduced by RNAi in IPCs (Figure 5). We further tested whether *Upd2*, a human *Leptin* homolog in *Drosophila* [41], is affected by *dSec16* RNA in IPCs. A higher mRNA level of *Upd2* was seen when compared to the control flies (Figure 5). Moreover, the adipokinetic hormone (AKH) plays a vital role in lipid metabolism by functioning as a glucagon-like endocrine [42]. Our result showed that the expression of AKH was decreased in flies with *dSec16* RNAi in IPCs (Figure 5). Altogether, these results indicated that the knockdown of *dSec16* in IPCs regulates energy metabolism by modulating the expression of endocrine peptides in adult flies.

## 4. Discussion

*dSec16* is a peripheral membrane protein involved in the biological processes of coat protein II (COPII) formation, which is considered a crucial responsive physiological process in energy balance [28]. *dSec16* is a *Drosophila* homolog of human *SEC16B*, which was identified as an obesity gene in multiple GWASs but was not functionally validated. Our results showed that *dSec16* played an important role in energy balance regulation in flies. The global knockdown of *dSec16* resulted in hyperphagia and fat accumulation. We further demonstrated that *dSec16* acting in the nervous system, primarily in IPCs, controls fat accumulation. In addition, *dSec16b* acting in IPCs affected the expression of endocrine peptides from IPCs, corpora cardiaca cells (CC cells) and fat body, providing a novel perspective of a secretory-related gene influencing energy balance. In this study, we, for the first time, show the physiological role of *dSec16* in the regulation of energy metabolism in flies, suggesting a potential gene in human obesity.

Using TAG as a main energy storage form is an efficient and flexible strategy that is evolutionarily conserved. However, excessive fat accumulation can lead to common metabolic diseases including non-alcoholic fatty liver disease and obesity [43]. In *Drosophila*, IPCs produce and secrete the insulin-like peptides (Ilps) [44], which function as mammalian insulin in controlling fat accumulation [45]. Insulin signaling activation in fat body, a tissue similar to mammalian white adipose tissue and liver, inhibits TAG decomposition and promotes TAG storage [46]. In this study, reducing *dSec16* mRNA expression in IPCs increased TAG levels, suggesting that *dSec16* affects fat accumulation possibly by interfering with the release of dIlps from IPCs. The finding that dSec16 affected the expression of *dIlp2* and *dIlp3* but not *dIlp5* was partially consistent with a previous study [47]. Our finding of the downregulation of *Akh* in flies with *dSec16b* knockdown in IPCs is consistent with that AKH antagonizes insulin signaling and promotes utilization of TAG. Similarly, we observed an upregulation of *Upd2* with increased TAG storage, as it has been proven that *Upd2* promotes dIlp2 and dIlp5 secretion by the IPCs [41]. Nevertheless, *Upd2,* a fly homolog of human leptin, does not influence feeding [41]. Our data showed that food intake was not increased in IPCs knockdown as global knockdown, indicating that food intake was possibly regulated by *dSec16* functions in cholinergic neurons (*Chat-Gal4*) and dopaminergic neurons (*DDC-Gal4*). Further studies are required to investigate the role of *dSec16* in the regulation of insulin signaling.

Similar to mammals, *Drosophila* dIlps are released in response to high levels of circulating sugars, while AKH is released in response to low levels of circulating sugars [48]. In our study, the knockdown of *dSec16* in IPCs results in insufficient dIlps and decreased expression of *AKH*, indicating a high level of circulating sugars that trigger fuel storage [49]. Glycogen is another indispensable energy storage form in flies [50]. Pan-neuronal *dSec16* knockdown promoted the resistance towards starvation under HSD. This could be explained by the utilization of not only TAG but also glycogen to meet nutrient needs.

Our data showed that pan-neuronal *dSec16* knockdown had different effects on food under ND and HSD, and these effects were different from that of global *dSec16* knockdown. This suggests that the role of *dSec16* in food intake is dependent on tissues and diets. Neuronal insulin signaling is important for food intake [51]. In rodents, insufficient insulin signaling in pan-neurons increased food intake in a normal diet [52]. It has been established that *Drosophila* showed hyperphagia under HSD feeding [53]. HSD can enhance insulin signaling in *Drosophila* [54]. Prolonged HSD triggers hyperglycemia and insulin resistance, which increases feeding in *Drosophila* [55,56]. However, it has been shown that although HSD induces insulin resistance through dIlps compensation, after sustained HSD, dIlps compensation is no longer effective, in which case *Drosophila* develops hyperglycemia leading to hyperphagia [57]. In addition, neuronal signaling including octopaminergic, dopaminergic and neuropeptide F signaling regulates feeding by interacting with insulin signaling [39]. Our data showed that *dSec16,* specifically RNAi in dopaminergic neurons, significantly decreased food intake. This suggests that dSec16 acts in dopaminergic neurons controlling food intake, however, the underlying mechanism still needs to be investigated.

Except for the nervous system, fat body [58] and muscles [59] are also involved in the regulation of energy homeostasis and there are complex cross-talks among these organs. A previous study demonstrated that the overexpression of ERK7 in fat body but not IPCs inhibited lipid accumulation in *Drosophila* [60]. The IPCs-specific *dSec16* RNAi could not complete phenocopy metabolic phenotypes caused by global *dSec16* RNAi, indicating *dSec16* may function in other tissues controlling energy homeostasis. Further research to confirm if *dSec16* functions in these tissues are required.

Overall, we identified *dSec16* as a regulator of energy homeostasis in *Drosophila*. Our finding implies that *dSec16* is potentially linked to metabolic diseases such as obesity. Our study provides a new model for investigating the function of obesity GWAS genes in energy balance and advances the molecular understanding of obesity.

## Data Availability

Not applicable.

## Supplementary Materials

The following supporting information can be downloaded at: https://www.mdpi.com/article/10.3390/life13010081/s1, Figure S1: Relative dSec16 expression in flies with or without RU486 activation in whole Drosophila body. n, indicates a biological replicate of 15 flies. Data are presented as mean ± SEM. Two-tailed unpaired *t*-test was used. ** *p* < 0.01; Figure S2: (A–C) RU486 had no effect on food intake (H, *n* = 10 replicate), body weight (I, *n* = 6 replicate), or TAG (J, *n* = 6 replicate). All experiments were done in male adult flies (4–7 days old) fed on a normal diet. n, indicates a biological replicate. Data are presented as mean ± SEM. Two-tailed unpaired *t*-test was used. * *p* < 0.05, ** *p* < 0.01, *** *p* < 0.001, **** *p* < 0.0001; Table S1: List of primers used for the RT-QPCR analysis. All primers are displayed in 5’-3’ direction.
