# Supplementary material for: dSec16 Acting in Insulin-like Peptide Producing Cells Controls Energy Homeostasis in Drosophila
Source: Life (Basel). 2022 Dec 27;13(1):81. doi: 10.3390/life13010081 (PMC9862641; doi:10.3390/life13010081)
Supplement: Supplementary file 1 [file life-13-00081-s001.zip › life-1963151-supplementary.pdf]

dSec16* Acting in Insulin-Producing Cells Controls Energy Homeostasis in *Drosophila

Ruo-Xin Zhang[1], Sha-Sha Li[1], An-Qi Li[1], Zhi-Ying Liu[1], Qiao-Ping Wang[1*]

*Correspondence: wangqp7@mail.sysu.edu.cn(Q.P.W.)

This PDF file includes:

Figs. S1 to S2
Table S1

Supplementary Material

Figure S1

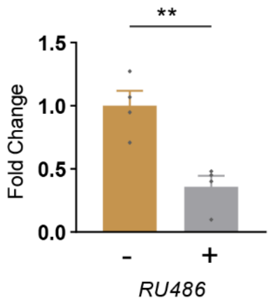

Figure S1

Relative *dSec16* expression in flies with or without RU486 activation in whole Drosophila body. n, indicates a biological replicate of 15 flies. Data are presented as mean ± SEM. Two-tailed unpaired t-test was used. **$p < 0.01$.

Figure S2

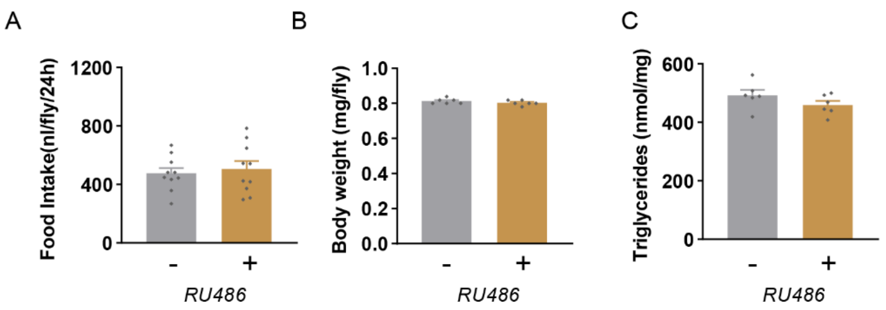

Figure S2

(A-C) RU486 had no effect on food intake (H, n=10 replicate), body weight (I, n=6 replicate), or TAG (J, n=6 replicate). All experiments were done in male adult flies (4-7 days old) fed on a normal diet. n, indicates a biological replicate. Data are presented as mean ± SEM. Two-tailed unpaired t-test was used. *p < 0.05, **p < 0.01, ***p < 0.001, ****p < 0.0001.

Abbreviation	Primer Pairs
rp49	5’-ATCGGTTACGGATCGAACAA-3’<br>5’-GACAATCTCCTTGCGCTTCT-3’
dilp2	5’-AGCAAGCCTTTGTCCTTCATCTC-3’<br>5’-ACACCATACTCAGCACCTCGTTG-3’
dilp3	5’-TGTGTGTATGGCTTCAACGCAATG-3’<br>5’-CACTCAACAGTCTTTCCAGCAGGG-3’
dilp5	5’-GAGGCACCTTGGGCCTATTC-3’<br>5’-CATGTGGTGAGATTCGG-3’
upd2	5’-CACAAGTGCGGTGAAGCTAA-3’<br>5’-GGCTCTTCTGCTGATCCTTG-3’
AKH	5’-AGACCTCCAACGAAATGCTG-3’<br>5’-GTGCTTGCAGTCCAGAAAGAG-3’
dSec16	5’-GCGGACGCTACAACTA-3’<br>5’-GCAGGATCTGCCATTA-3’

Table S1.

List of primers used for the RT-QPCR analysis. All primers are displayed in 5’-3’ direction.
